# A Theoretical Study on Terpene‐Based Natural Deep Eutectic Solvent: Relationship between Viscosity and Hydrogen‐Bonding Interactions

**DOI:** 10.1002/gch2.202000103

**Published:** 2021-01-12

**Authors:** Chen Fan, Yang Liu, Tarik Sebbah, Xueli Cao

**Affiliations:** ^1^ Beijing Advanced Innovation Center for Food Nutrition and Human Health Beijing Technology and Business University No. 11 Fucheng Road Beijing 100048 China

**Keywords:** deep eutectic solvents, hydrogen bonding interactions, quantum chemistry, terpenes, viscosity

## Abstract

The aim of this work is to shed light on the origins of unique properties by studying the relationship between viscosity and hydrogen‐bonding interactions of terpene‐based natural deep eutectic solvents (NADES). Five systems including camphor/formic acid, menthol/acetic acid, menthol/β‐citronellol, menthol/lactic acid, and thymol/β‐citronellol are prepared (molar ratio 1:1). Their structures and nature of the associated hydrogen bonds are investigated through multiple methods and theories. The viscosity of NADES is consistent with the product of hydrogen‐bond number and lifetime. Through visualization of non‐covalent interactions, terpene‐acid‐based NADES with single sites show the lowest viscosity among the studied systems because of weak and unstable hydrogen bonding. Inversely, multi‐site terpene‐acid‐based NADES possess relatively high viscosity. Owing to the stability of hydrogen bonds in the network, the terpene‐terpene‐based system is in the middle level of viscosity. In‐depth analysis of these hydrogen bonds shows that they can be classified as “weak to medium” and are mainly derived from electrostatic interactions. Moreover, there is an obvious connection between viscosity and hydrogen‐bonding strength (integrated core‐valence bifurcation index) in the networks. The discovery of intrinsic rules between viscosity and hydrogen‐bonding interactions is beneficial for the design of novel low‐viscosity NADES in the future.

## Graphical abstract

The structures and inner nature of the hydrogen‐bond network of different systems are studied by multiple methods. The discovery of intrinsic rules between viscosity and hydrogen‐bonding interactions is beneficial for the rational and effective design of novel low‐viscosity natural deep eutectic solvents.

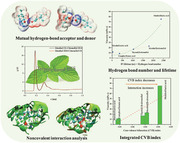

## 1 Introduction

The risks and dangers of chemical residues connected to human health and environmental issues are a concern.^[^ ^1^ ^]^ A crucial element for the sustainable chemical processes is based on the development of novel green agents and solvents.^[^ ^2^ ^]^ At the beginning of this century, deep eutectic solvent (DES) attracted attention as a new subclass of ionic liquid (IL) because of similar characteristics and properties.^[^ ^3^, ^4^ ^]^ However, they have been gradually shown to be a totally different class of liquids. DES is composed of hydrogen bond donors (HBDs) and acceptors (HBAs) through weak‐force interactions.^[^ ^5^ ^]^ They are usually described as hydrogen‐bonding complexes even if the existence of hydrogen bonds cannot offer conditions to define them.^[^ ^6^ ^]^ DES has become a large family of potentially more sustainable alternative solvents.^[^ ^7^ ^]^ Natural products or compounds were introduced to DES for better environmental impacts with low toxicity and high biodegradability.^[^ ^8^, ^9^ ^]^ This recently discovered category was named natural DES (NADES), which has aroused great interest in many fields such as analytical chemistry,^[^ ^10^, ^11^ ^]^ catalysis,^[^ ^12^ ^]^ biomass pretreatment,^[^ ^13^ ^]^ material science,^[^ ^14^, ^15^, ^16^ ^]^ and drug delivery.^[^ ^17^ ^]^

The viscosity of DES plays an important role in determining how they are used industrially. High viscosity will strongly affect the mass transport phenomena and heat transfer rate as well as the conductivity of solvent. It hinders the mobility of dissolved species. The viscosity of the widely studied choline chloride (ChCl)‐based NADES ranged from 50 mPa·s to as high as 2000 Pa·s at ambient conditions. It is higher than that of imidazolium‐based IL.^[^ ^18^ ^]^ ChCl‐based NADES is classified as ionic DES. The high viscosity of this kind of DES is mainly due to strong electrostatic interactions, small void volume, large ion size, and free volume.^[^ ^19^, ^20^ ^]^ In ionic and non‐ionic DES, the viscosity increases as chain length of cation alkyl or organic acid (HBD).^[^ ^21^, ^22^ ^]^ Another important factor is the water content that can influence the viscosity of DES since numerous types of eutectic mixtures are highly hygroscopic.^[^ ^19^ ^]^ Generally, the viscosity of the eutectic mixture depends on the chemical nature of DES components (HBD and HBA).^[^ ^23^ ^]^

The first reported hydrophobic DES is based on quaternary ammonium salts.^[^ ^24^ ^]^ Hydrophobic eutectic solvents can lead to the utilization of eutectic mixture in applications that need direct contact with water. The concept of terpene‐based hydrophobic NADES was introduced by Marrucho's group in 2015 when they combined menthol with carboxylic acids.^[^ ^25^ ^]^ Terpenes or terpenoids are a large class of organic hydrocarbons (more than hundreds) found in many plants and insects. Since then, terpenes and diverse natural renewable resources forming hydrophobic NADES systems have been developed.^[^ ^26^, ^27^, ^28^ ^]^ Despite the significant interest of this novel class of DES, the number of proposed systems is very limited so far.^[^ ^29^ ^]^ Moreover, these hydrophobic solvents usually possess relatively low viscosity. They are composed of neutral compounds, and thus intermolecular interactions are responsible for the formation of a network that restricts the mobility of free species.^[^ ^29^ ^]^ Their eutectic property stems from hydrogen bonds established between HBA and HBD, which are much stronger than that in any of the pure components.^[^ ^30^ ^]^ Therefore, the viscosity of terpene‐based NADES is closely linked to hydrogen‐bonding interactions in the systems. However, a general relationship between hydrogen bonds and viscosity of terpene‐based NADES still remains unclear and is the cornerstone of effectively designing novel low‐viscosity NADES.

Herein, molecular dynamics (MD) simulations and quantum mechanical calculations (QC) were used to explore the relationship between viscosity and hydrogen‐bonding interactions of five terpene‐based NADES systems including camphor/formic acid, menthol/acetic acid, menthol/β‐citronellol, menthol/lactic acid, and thymol/β‐citronellol. Structures of systems, properties of non‐covalent interactions (NCIs) as well as the number, lifetime, strength, and nature of hydrogen bonds were systematically studied. This study aims to investigate various complex factors influencing the viscosity to better understand the origins of unique properties of the hydrogen‐bonding liquids and to aid in the promotion of developing NADES with low viscosity.

## 2 Results and Discussion

### 2.1 Radial and Spatial Distribution Functions

**Figure 1:**
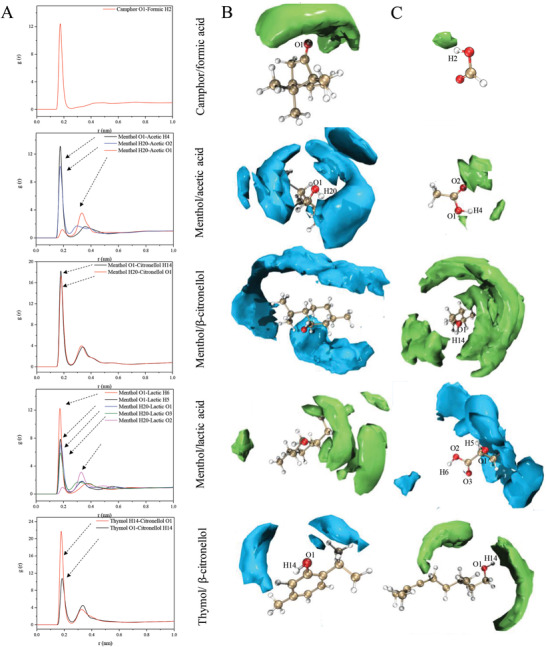
Radial and spatial distribution functions (RDF and SDF) of different natural deep eutectic solvent at 1 bar and 298 K. A) Atom–atom radial distribution functions; B,C) Hydrogen bond donor (HBD)‐hydrogen bond acceptor (HBA) spatial distribution functions. The surfaces represent isosurfaces of normalized concentration for mass center of different components (blue or green).

The densities of all NADES were first calculated to test the accuracy of the used force field. As shown in **Table** **1**, all calculated results are consistent with the experimental densities (error less than 3%). This indicates that the employed all‐atom general AMBER force field (GAFF) is suitable for the five NADES systems. All systems were investigated in terms of atom–atom radial distribution functions (RDFs) and spatial distribution functions (SDFs) (**Figure** **1**). Term *g*(*r*) is a measure of the probability that an atom will be located a distance from another atom. In Figure 1A, all investigated peaks for RDF between the oxygen and hydrogen atoms of target part are located at near 2 Å, which presumably refer to the intermolecular hydrogen‐bonding forces for the relatively simple systems.^[^ ^31^ ^]^ Peaks at or greater than 0.3 nm are probably non‐hydrogen bonded intermolecular interactions and do not contribute to the hydrogen‐bonding network.^[^ ^31^ ^]^ The intensity of some peaks is weak, suggesting that these atom–atom interactions do not play significant roles in the solvation of target systems.^[^ ^30^ ^]^

**Table 1:** Comparison between experimental and simulated density data at 1 bar and 298 K

Deep eutectic solvent	Molar ratio	ρ^exp^ [g cm^–3^]	ρ^sim^ [g cm^–3^]	Deviation [%]
Menthol/lactic acid	1:1	0.988	0.984	0.41
Menthol/acetic acid	1:1	0.931^a)^	0.954	2.47
Camphor/formic acid	1:1	1.000	1.009	0.90
Menthol/β‐citronellol	1:1	0.873	0.880	0.80
Thymol/β‐citronellol	1:1	0.911	0.913	0.22

^a)^ Obtained from reference.^[^ ^25^ ^]^

For camphor/formic acid system, only one peak for camphor O1‐formic H2 at around 0.2 nm demonstrates that the proton hydrogen of the acid shows strong interactions. For the menthol/acetic acid system, the probability of menthol O1‐acetic H4 is slightly larger than that of menthol H20‐acetic O2. This indicates that menthol is prone to act as HBA in this system. Menthol O1‐citronellol H14 and menthol H20‐citronellol O1 have almost the same chance to form hydrogen bonds in the menthol/β‐citronellol system.

In our previously published work, menthol or thymol was proven to interact with other constituents in eutectic mixtures as both HBA and HBD.^[^ ^32^ ^]^ Menthol, thymol, and β‐citronellol (all natural small molecules and terpenoids) can undoubtedly act as mutual HBAs and HBDs in terpene‐terpene based NADES systems. For thymol/β‐citronellol system, the intensity of the RDF in thymol H14‐citronellol O1 is slightly stronger than that of thymol O1‐citronellol H14 due to the strong electrophilic effect of the benzene ring. Versus the above single‐site eutectic mixtures, the menthol/lactic acid system has multiple sites. The hydroxyl oxygen atom of menthol connecting carboxyl hydrogen atom is the most noticeable interaction, and it presents similar features as the menthol/acetic acid system. However, menthol O1‐lactic H5 and menthol H20‐lactic O1 have almost the same probability, suggesting that there is typical dual‐site phenomenon in this kind of eutectic mixture. As a result of complex hydrogen‐bonding interactions, it has the largest viscosity of the five systems.

The coordination numbers of the NADES systems were calculated based on RDF: 0.42 for menthol/lactic acid, 0.34 for menthol/acetic acid, 0.24 for camphor/formic acid, 0.27 for menthol/β‐citronellol, and 0.33 for thymol/β‐citronellol. These mixtures are not only formed at a given stoichiometric proportion (usually connected to complex formation) but in a mole fraction range for individual system.^[^ ^6^ ^]^ To visualize the distribution of HBA or HBD around the other component in NADES system, the SDF results are also plotted in Figure 1B,C. They are almost consistent with the above‐mentioned RDF results and intuitively show the types and distributions of interaction sites in the entire NADES systems.

### 2.2 Hydrogen‐Bond Number and Lifetime

**Figure 2:**
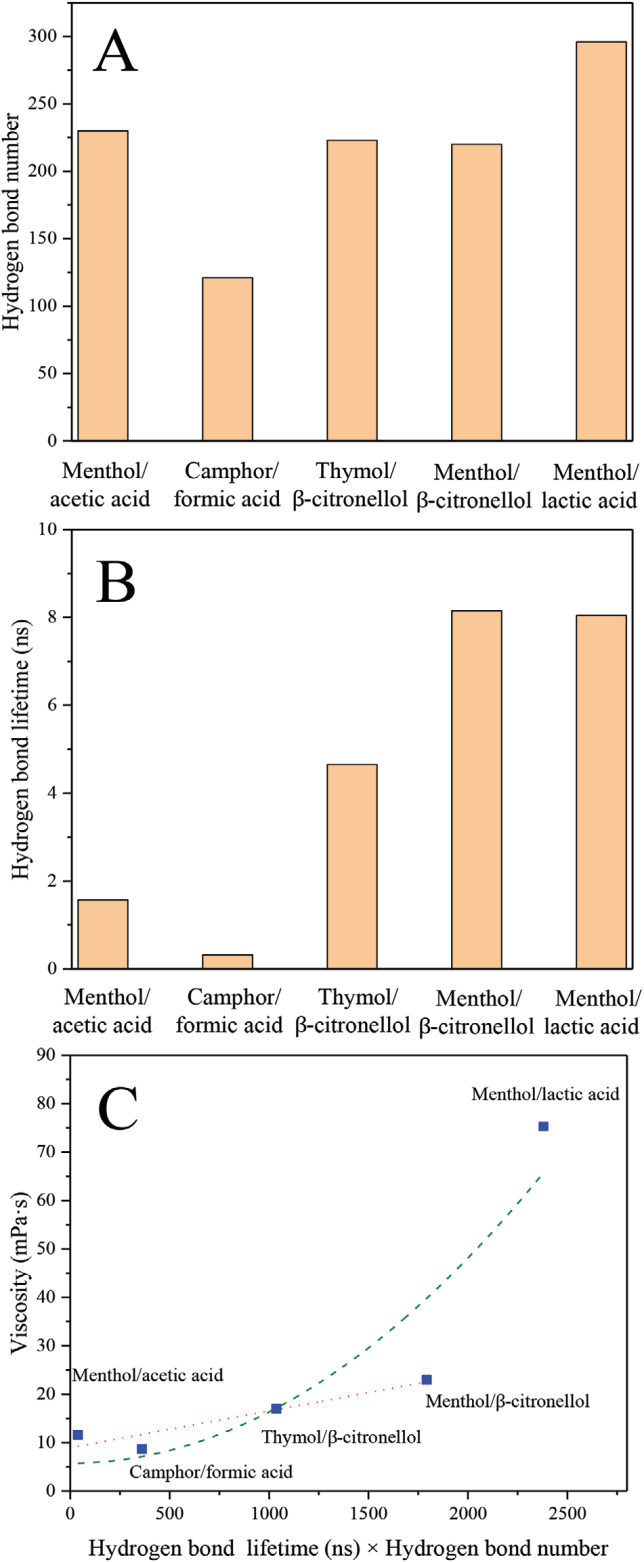
Hydrogen‐bonding interactions analysis of different natural deep eutectic solvents at 298 K. A) Hydrogen‐bond number; B) hydrogen‐bond lifetime; and C) correlation between the experimental viscosity and the product of hydrogen‐bond number and lifetime.

The hydrogen‐bond number and lifetime were studied to further investigate the relationship between viscosity and hydrogen‐bonding interactions in terpene‐based NADES. The calculated lifetime based on stable states pictures (SSPs) includes unsuccessful H‐bond exchanges, which can describe the hydrogen‐bonding network more realistically. The hydrogen bond relaxation is a hydrogen bond exchanging process, which means that the labeled hydrogen switches its HBA from O^a^ to O^b^ (oxygen atom a and b).^[^ ^33^ ^]^ Calculation procedures were used according to previously published works.^[^ ^33^, ^34^ ^]^ **Figure** **2**A shows that the average hydrogen‐bond number decreases on the order of menthol/lactic acid > menthol/acetic acid > thymol/β‐citronellol > menthol/β‐citronellol > camphor/formic acid, suggesting that lactic acid‐based system has multiple interaction sites. Additionally, hydrogen‐bond lifetime increases on the order of camphor/formic acid < menthol/acetic acid < thymol/β‐citronellol < menthol/lactic acid < menthol/β‐citronellol, which is inconsistent with the viscosity of investigated systems (Figure 2B). According to the literature, the relationship between lifetime of hydrogen bonds and viscosity of ILs nearly followed a linear correlation.^[^ ^35^ ^]^ Therefore, they are quite different at this point. Moreover, the average numbers of H‐bond per DES molecule were obtained: 1.21 for menthol/lactic acid, 0.94 for menthol/acetic acid, 0.49 for camphor/formic acid, 0.88 for menthol/β‐citronellol, and 0.89 for thymol/β‐citronellol.

The viscosity of terpene‐based NADES increases with the product of the number and lifetime of H‐bonds (Figure 2C). Furthermore, for single‐site eutectic mixtures, the relationship between viscosity and product of two factors has an excellent linear correlation. This means that the number and lifetime synthetically influence the viscosity of NADES. The types of systems (single or multiple sites) decide the number of hydrogen bonds. Meanwhile, the lifetime is traditionally considered an important element to evaluate the strength of the H bond.^[^ ^34^ ^]^

### 2.3 Averaged NCIs

**Figure 3:**
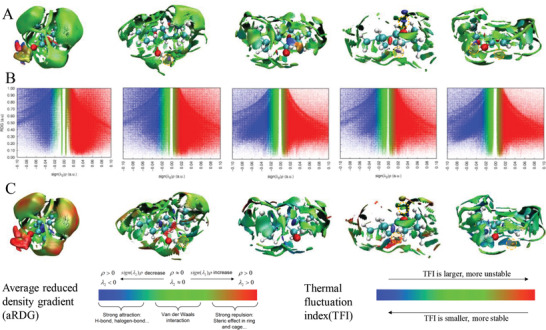
Visualization and analysis of averaged non‐covalent interaction (aNCI) in different natural deep eutectic solvents at 298 K. A) Averaged non‐covalent interaction analysis; B) color mapped scatter plots of the reduced density gradient versus the electron density; C) fluctuation index (TFI). The color mapped surfaces represent isosurfaces of averaged reduced density gradient with an isovalue of 0.25.

The NCI method, also known as reduced density gradient (RDG) method, is a valuable tool for studying the properties of weak interactions.^[^ ^36^ ^]^ The averaged NCI (aNCI) index was developed for a thermal fluctuation environment.^[^ ^37^ ^]^ In Bader's atoms in molecules theory,^[^ ^38^ ^]^ the NCI strength has positive correlation with electron density (ρ). **Figure** **3** (left) shows that highly attractive interactions have a relatively large ρ (blue region) such as hydrogen bonds and halogen bonds. Weak interactions have low ρ (green region) such as dispersive‐like van der Waals interactions, and repulsive interactions also have a large ρ (red region) such as steric clashes. More blue indicates the hydrogen‐bonding interaction or electrostatic effect in certain regions is stronger. The aNCI method also defines a new quantity named the thermal fluctuation index (TFI) to reveal the stability of NCIs.^[^ ^37^ ^]^ More blue or red means that the TFI is smaller or larger; hence, the interactions in the corresponding region are more stable or unstable (illustration of Figure 3 on the right).

To conveniently identify the relevance between spikes and RDG isosurfaces, 2D scatter plots were mapped via the corresponding color. Figures of the RDG value versus ρ for these NADES systems all exhibit several spikes in the low‐density and low‐gradient regions, which indicate a signature of NCIs in NADES (Figure 3B). The visualized aNCI and TFI indexes of different systems are depicted in Figure 3A,C, respectively. For the camphor/formic acid system (the simplest one among five NADES), a relatively strong hydrogen bond is formed, and there is a repulsive interaction zone around the camphor O1. When a hydrogen‐bond network forms, the hydrogen atom of the formic acid can interact with the oxygen atom of camphor from any direction. This fluctuation is reflected in the large TFI value. The instability of camphor acting as HBA is partially responsible for its low shear viscosity. The hydrogen bond formed by menthol as HBD is observed is stronger than that when HBA is in a menthol/acetic acid system. The lowest fluctuations are encountered around the HBD interactions region, which means that the HBD performance of menthol is mostly stable. The low viscosity of menthol/acetic acid system is probably attributed to the weak and unstable bond type that mainly exists in the H‐bond network.

For the menthol/β‐citronellol system, the hydrogen bond formed by menthol as HBD is stronger than that of HBA. Unlike the menthol/acetic acid system, the relatively high fluctuations are encountered around the HBD interaction region, which illustrates that menthol O1 is more stable in constructing hydrogen bonds (half blue region). The analogous hydrogen‐bond donning and accepting capability can be found in the thymol/β‐citronellol system. However, it is obvious that the hydrogen bond formed by thymol as HBA is more flexible than that of HBD in this system. The interaction network stability of terpene‐terpene based NADES is generally higher than that of the terpene‐acid‐based NADES among the studied single‐site systems, which leads to higher viscosity. In a multi‐site menthol/lactic acid system, the resulting hydrogen bond is strong (dark blue region) when either menthol acts as HBA or HBD. Both systems have some stability. The interaction energies among various components of NADES from the MD simulation data were also calculated (ranged from 46.37 to 81.18 KJ mol^–1^). It can be seen in Figure S1, Supporting Information, that they are inconsistent with the viscosities of investigated systems.

### 2.4 Quantum Chemistry Calculations

QC was conducted to further investigate the hydrogen‐bonding ability of different types. **Table** **2** presents the binding energy (BE) values of eleven hydrogen bonds. A new and rigorous classification of hydrogen bonds based on a symmetry‐adapted perturbation theory decomposition was proposed in 2019.^[^ ^39^ ^]^ It sheds light on the nature of hydrogen bonds of different types and strengths. In this method, the magnitude of BE is straightly correlated with its dominating physical properties. Neutral complexes either belong to a “very weak” type with a BE value larger than −2.5 kcal mol^–1^ that is mainly dominated by two interactions (dispersion and electrostatic forces) or to “weak‐to‐medium” type with BE between −2.5 and −14.0 kcal mol^–1^ that is merely dominated by electrostatics. Furthermore, charged complexes are classified as “medium” category with BE from −11.0 to −15.0 kcal mol^–1^ (mainly derived by electrostatic interactions) or as “strong” category with BE less than −15.0 kcal mol^–1^ (derived by electrostatic and induction forces). The BE values of all hydrogen bonds varied from −4.75 to −.95 kcal mol^–1^ and can be put into the “weak‐to‐medium” category. This illustrates that the formation of hydrogen bond is dominated by electrostatic interaction in terpene‐based NADES systems.

**Table 2:** Binding energy of the studied complexes computed at the B3LYP/ma‐def2‐TZVP level with counterpoise correction

Deep eutectic solvent	Hydrogen bond	Binding energy [kcal mol^–1^]
Camphor/formic acid	Camphor O1‐formic H2	−5.75
Menthol/acetic acid	Menthol O1‐acetic H4	−7.20
	Menthol H20‐acetic O2	−8.28
Menthol/β‐citronellol	Menthol O1‐citronellol H14	−4.80
	Menthol H20‐citronellol O1	−5.90
Menthol/lactic acid	Menthol O1‐lactic H5	−7.21
	Menthol O1‐lactic H6	−9.95
	Menthol H20‐lactic O1	−4.75
	Menthol H20‐lactic O3	−5.21
Thymol/β‐citronellol	Thymol O1‐citronellol H14	−6.39
	Thymol H14‐citronellol O1	−7.28

**Figure 4:**
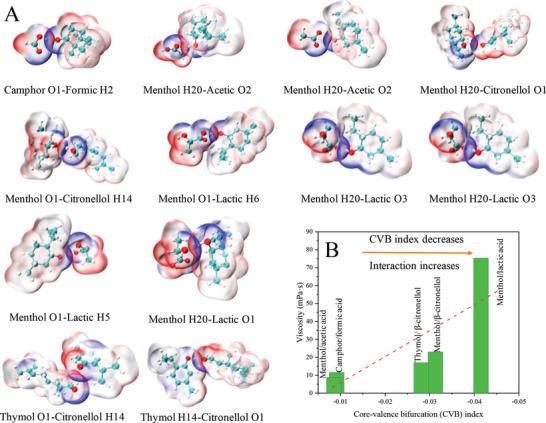
A) Molecular surface colored by electrostatic potential surface (ESP) for mutual penetration; B) correlation between the experimental viscosity and integrated core‐valence bifurcation (CVB) index. The surfaces represent isosurfaces with high electron density regions (HBA sites) in blue and low electron density regions (HBD sites) in red.

The electrostatic potential surface (ESP) mapped surface is shown in **Figure** **4**A. Mutual penetration occurs between the surfaces of HBA and HBD because of the formation of hydrogen bonds. In addition, the mapped colors show that bonding systems are formed in a complementary way by ESP positive‐negative connections revealing the electrostatic nature of the hydrogen bonds. This coincided with the results of BE analysis.

A core‐valence bifurcation (CVB) index was defined based on electron localization function (ELF) and is mainly used to elaborately distinguish various hydrogen bonds.^[^ ^40^ ^]^ A typical H‐bond form (D‐H⋅⋅⋅A) is expressed as CVB index = ELF (C‐V) − ELF (DH‐A) where D = donor, H = hydrogen, A = acceptor, and ELF(C‐V) stands for the ELF bifurcation value between ELF core domain and valence domain, while the ELF (DH‐A) corresponds to the ELF value at a bifurcation point between the donor hydrogen and the acceptor.

The CVB index of weak hydrogen bonds is positive and that of relatively strong hydrogen bonds is usually negative. It decreases as the strength of the interaction increases.^[^ ^41^ ^]^ To establish a simple and direct relationship between hydrogen‐bonding interactions and viscosity, the strength of the hydrogen bond in the network and the viscosity of the studied systems are plotted in Figure 4B. Integrated CVB indexes were calculated on the basis of listed hydrogen bond types. They successfully differentiate the H‐bond strength of five systems. There is an obvious connection between viscosity and the CVB index in the NADES network. A higher H‐bond strength of the network leads to a larger viscosity. On the one hand, multiple studies have tried to propose rules relating structural characteristics of molecules to viscosity of liquids.^[^ ^42^ ^]^ On the other hand, many complex items affect viscosity, and it is difficult to associate one certain micro‐structural factor to viscosity in a direct way.

## 3 Conclusion

In this work, MD simulations and QC were employed to investigate the relationship between viscosity and hydrogen‐bonding interactions of terpene‐based NADES. The structures and inner nature of hydrogen‐bond network of different systems were studied by multiple methods and theories including RDF, SDF, aNCI, and CVB. According to structure analysis, five NADES can be roughly divided into single‐site and multi‐site systems. At the same time, there are terpene‐acid based NADES and terpene‐terpene based NADES in single‐site systems. Their viscosity is quite consistent with the product of hydrogen‐bond number and lifetime. The two factors synthetically influence the viscosity of eutectic mixtures. Through visually studying NCIs in the structural network, the low viscosity of terpene‐acid‐based single‐site NADES is mainly attributed to weak and unstable H‐bond type. The interactions in terpene–terpene‐based NADES are more stable than those in terpene‐acid‐based systems. In‐depth analysis of these hydrogen bonds showed that they could be classified as “weak to medium” and was mainly derived from electrostatic interactions. There is also an obvious connection between viscosity and H‐bond strength in NADES network. The discovery of intrinsic rules between viscosity and hydrogen‐bonding interactions is beneficial for the design of novel low‐viscosity NADES in the future.

## 4 Experimental Section

##### Preparation Methodology and Properties Measurements

**Figure 5:**
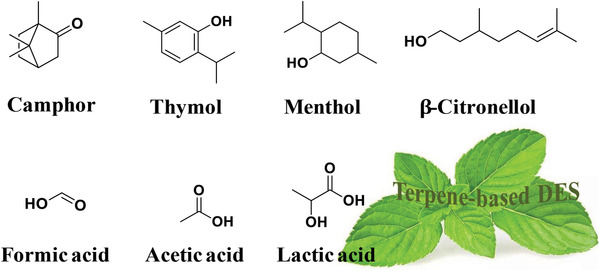
Terpenes and organic acids used for the preparation of natural deep eutectic solvent.

Camphor (purity 95%, melting point 179 °C, SciFinder), menthol (99%, 41–43 °C), thymol (97%, 52 °C), β‐citronellol (92%, <25 °C), lactic acid (92%, 16.8 °C), acetic acid (99%, 16.6 °C), and formic acid (98%, 8.4 °C) were used as HBA and HBD, which bought Heowns Biochemical Technology Co., Ltd. (Tianjin, China). Their structures are shown in **Figure** **5**. NADES systems were prepared by mixing them at a 1:1 molar ratio. The mixture was constantly stirred at 80 °C until the formation of a homogeneous transparent liquid. Finally, five systems could be obtained as liquids at room temperature. The viscosities and densities of the NADES systems were measured at 25 °C and atmospheric pressure using a NDJ‐8S digital rotational viscometer and MDJ‐600G densitometer (density 0.0001 g·cm^–3^; viscosity 2%), respectively. The properties of menthol/acetic acid, menthol/lactic acid, and camphor/formic acid systems have been studied.^[^ ^19^, ^25^ ^]^ The Fourier transform infrared characterization of menthol/β‐citronellol and thymol/β‐citronellol systems were conducted to prove the presence of hydrogen‐bond network (Figures S2 and S3, Supporting Information). Their viscosities at different temperatures were also evaluated (Table S1, Supporting Information). Equimolar ratio was chosen according to the reported eutectic mixture, H‐bond interaction sites, and same conditions for QC‐MD simulations.

##### Quantum Chemistry Calculations

Geometries of all NADES components and complexes were fully optimized at the B3LYP/6‐311++g(d,p) level with Grimme's DFT‐D3(BJ) empirical dispersion correction by using ORCA 4.2.1 package.^[^ ^43^ ^]^ The BE of HBA and HBD was evaluated by single‐point energy calculation with ma‐def2‐TZVP basis set, which includes the diffuse character in the def2‐TZVP basis set.^[^ ^44^, ^45^ ^]^ The basis set superposition error was corrected by the employing counterpoise technique.^[^ ^46^ ^]^ The vibrational frequency calculations were also conducted to verify the true minima of the optimized geometries.

##### MD Simulations

All MD simulations were conducted with GROMACS 2019.5 package.^[^ ^47^ ^]^ Molecules were described by GAFF.^[^ ^48^ ^]^ ≈5 × 5 × 5 nm cubic boxes containing a certain number of HBA and HBD molecules were constructed for MD simulations and were generated by Packmol package^[^ ^49^ ^]^ (Table S2, Supporting Information). The periodic boundary conditions were applied for cubic boxes in all directions with a tolerance of 2.0 Angs. The initial configurations were then subjected to energy minimization using the steepest descent algorithm followed by the conjugate gradient method at 400 K.^[^ ^50^ ^]^ The systems were then annealed from 400 to 298 K for 5 ns with the Berendsen thermostat. Isothermal‐isobaric (NPT) and canonical (NVT) ensemble steps were used to further relax these systems. The temperature was controlled by employing the Nosé−Hoover thermostat and the pressure was maintained by the Parrinello–Rahman approach. The time step was set to 1 fs. The MD simulations runs of 10 ns were conducted for all systems at 1 bar and at 298 K with a non‐bonded interactions cutoff of 1.2 nm. In all cases, long‐range interactions were calculated by the particle‐mesh Ewald procedure with a grid spacing of 0.16 nm and interpolation order of 4.^[^ ^51^ ^]^ The hydrogen‐bond analyses were performed after ≈1 ns when equilibration was reached. Visually studying the weak interactions was done by Multiwfn package 3.7.^[^ ^52^ ^]^ Visualization of the results was performed by visual MD (VMD).^[^ ^53^ ^]^

## Conflict of Interest

The authors declare no conflict of interest.

## Supporting information

Supporting Information
