# Supplementary material for: A Theoretical Study on Terpene‐Based Natural Deep Eutectic Solvent: Relationship between Viscosity and Hydrogen‐Bonding Interactions
Source: Glob Chall. 2021 Jan 12;5(3):2000103. doi: 10.1002/gch2.202000103 (PMC7933815; doi:10.1002/gch2.202000103)
Supplement: Supplementary file 1 — Supporting Information [file GCH2-5-2000103-s001.pdf]

© 2021 Wiley-VCH GmbH

Global Challenges Open Access

Supporting Information

for *Global Challenges,* DOI: 10.1002/gch2.202000103

A Theoretical Study on Terpene-Based Natural Deep Eutectic Solvent: Relationship between Viscosity and Hydrogen-Bonding Interactions

*Chen Fan, Yang Liu, Tarik Sebbah, and Xueli Cao**

© Copyright 2020. WILEY-VCH GmbH.

Supporting Information

A theoretical study on terpene-based natural deep eutectic solvent: Relationship between viscosity and hydrogen-bonding interactions

*Chen Fan, Yang Liu, Tarik SEBBAH and Xueli Cao **

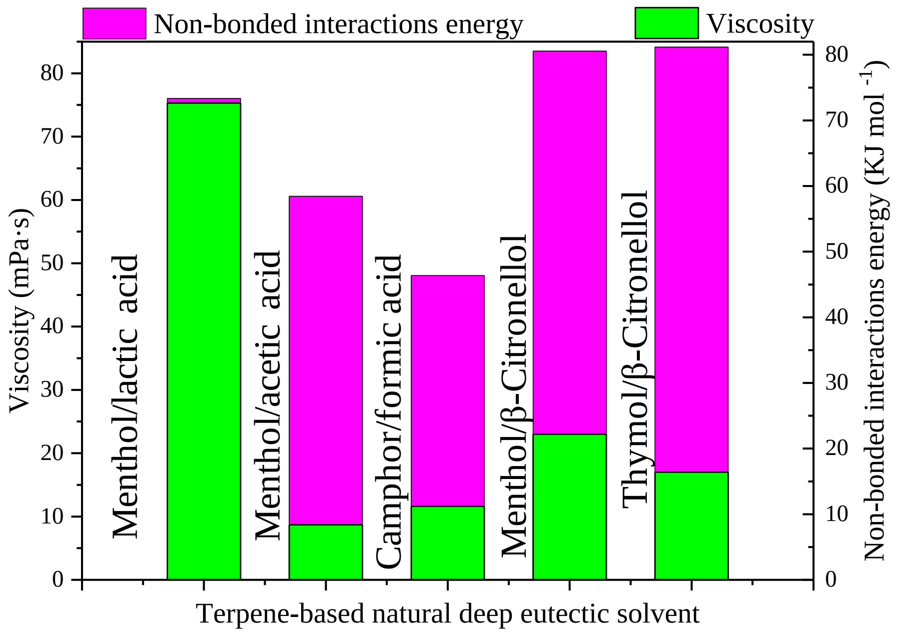

Figure S1. The interaction energies among various HBA and HBD components of NADES.

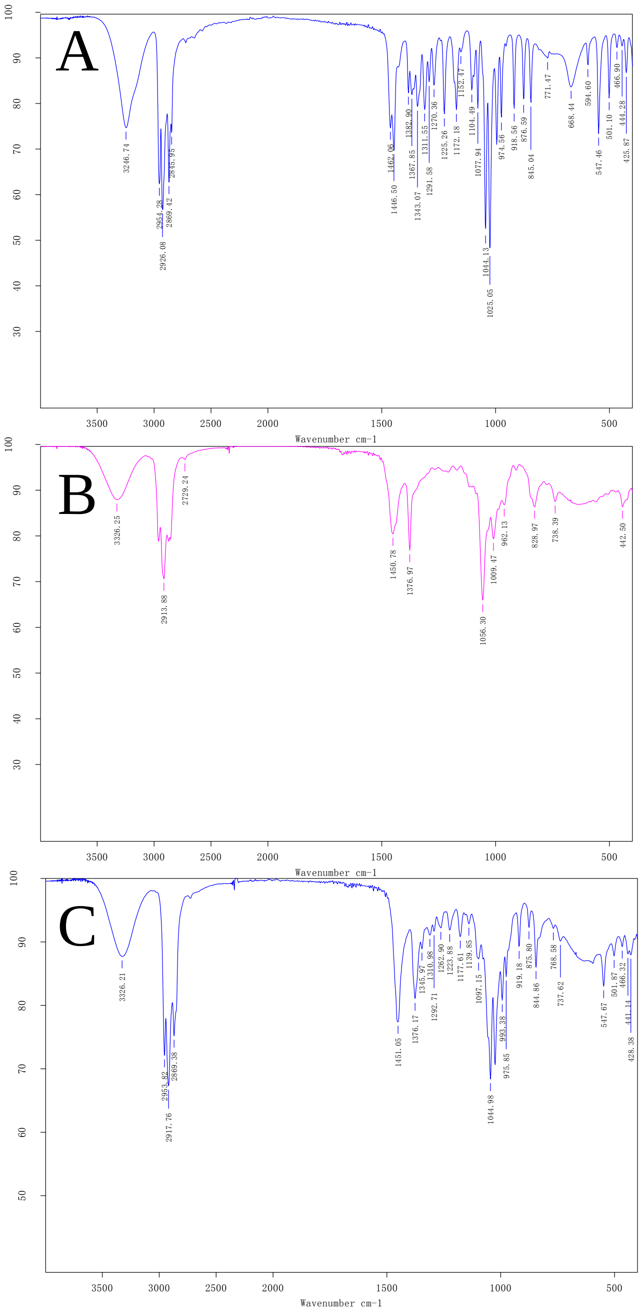

Figure S2. Fourier transform infrared spectra of menthol/β-citronellol based system. (A) Menthol; (B) β-Citronellol; (C) Corresponding natural deep eutectic solvent.

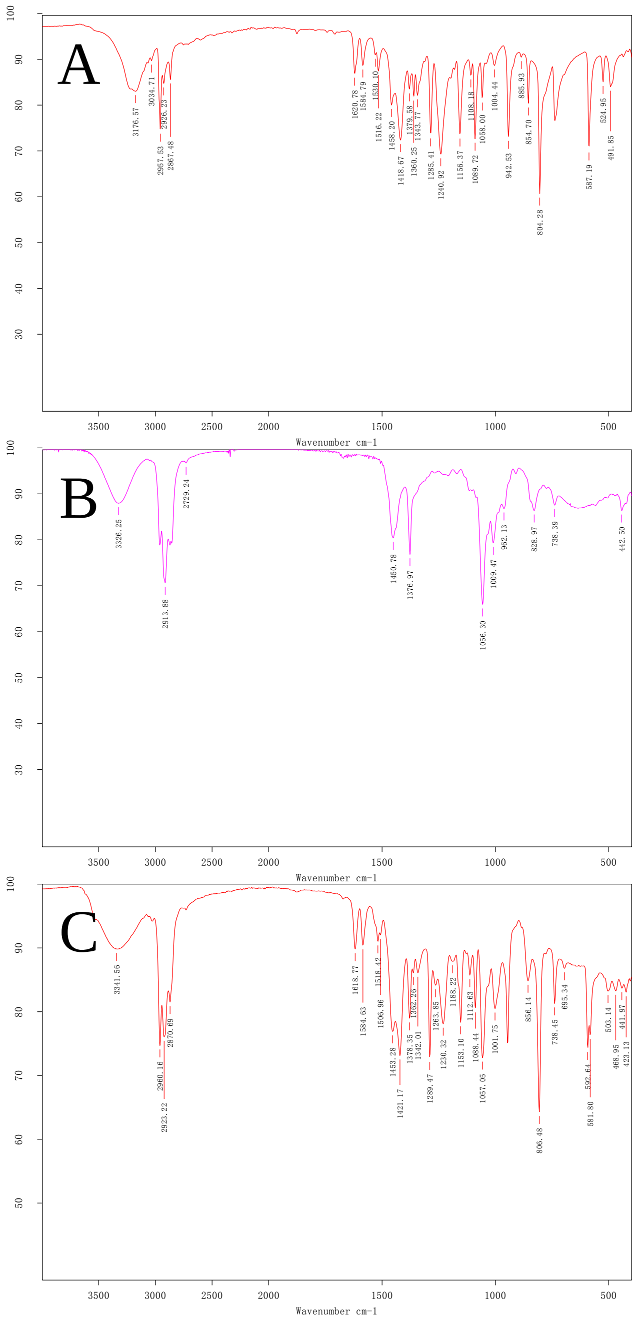

Figure S3. Fourier transform infrared spectra of thymol/β-citronellol based system. (A) Thymol; (B) β-Citronellol; (C) Corresponding natural deep eutectic solvent.

In Figure S1-S2, the Fourier transform infrared (FTIR) spectra of two NADES systems, it can be observed that stronger and broader bands (appeared between 3000 and 3500 cm^{-1}) indicate the presence of intermolecular hydrogen-bond network. On the other hand, in the FTIR spectra of NADES containing menthol, the hydroxyl band

from the menthol is originally located at lower wavenumber values (3246.74 cm^{-1}) and it shifts to the high values (3326.21^{-1}) in the eutectic mixtures. In the FTIR spectra of NADES containing thymol, the hydroxyl band from the thymol is originally located at lower wavenumber values (3176.57 cm^{-1}) and it shifts to the high values (3341.56^{-1}) in the eutectic mixtures. The shift of the hydrogen bond was due to the formation of NADES systems [1-2].

[1] A. R. Zarei, M. Nedaei, S. A. Ghorbanian, *J. Chromatogr. A* **2018**, *1553*, 32.

[2] D. J. Ramón, G. Guillena, Deep eutectic solvents: synthesis, properties, and applications, John Wiley & Sons, Inc., Hoboken, NJ**2020**.

Table S1. The viscosities of menthol/β-citronellol and thymol/β-citronellol systems at different temperature.

Deep eutectic solvent	Viscosity(mPa·S)						
	25 °C	35 °C	40 °C	45 °C	60 °C	70 °C	80 °C
Menthol:β-Citronellol	23.00	14.33	13.20	10.53	7.20	5.98	3.64
Thymol:β-Citronellol	17.00	12.40	8.35	7.18	5.80	5.25	2.96

Table S2. The molecule number and the optimized box size of simulated DES systems.

Deep eutectic solvent	Molecule number	Optimized box size
Menthol/lactic acid	250	4.63376 ×4.63376 ×4.63376
Menthol/acetic acid	250	4.50260 ×4.50260 ×4.50260
Camphor/formic acid	250	4.96037 ×4.96037 ×4.96037
Menthol/β-citronellol	250	4.97536 ×4.97536 ×4.97536
Thymol/β-citronellol	250	4.87573 ×4.87573 ×4.87573
